# Design of a Computerised Flight Mill Device to Measure the Flight Potential of Different Insects

**DOI:** 10.3390/s16040485

**Published:** 2016-04-07

**Authors:** Antonio Martí-Campoy, Juan Antonio Ávalos, Antonia Soto, Francisco Rodríguez-Ballester, Victoria Martínez-Blay, Manuel Pérez Malumbres

**Affiliations:** 1 Instituto de Tecnologías de la Información y Comunicaciones (ITACA), Universitat Politècnica de València, Camino de Vera s/n, 46022 Valencia, Spain; prodrig@disca.upv.es; 2 Instituto Agroforestal Mediterráneo (IAM), Universitat Politècnica de València, Camino de Vera s/n, 46022 Valencia, Spain; juavama@msn.com (J.A.Á); asoto@eaf.upv.es (A.S.); vicmarbl@etsia.upv.es (V.M.-B.); 3 Department of Physics and Computer Science, Miguel Hernandez University, Ave. Universidad s/n-Ed. Alcudia, 03202 Elche, Spain; mels@umh.es

**Keywords:** measurement, coleoptera, insect pests, flight study, flight mill, flight potential, behaviour, computer

## Abstract

Several insect species pose a serious threat to different plant species, sometimes becoming a pest that produces significant damage to the landscape, biodiversity, and/or the economy. This is the case of *Rhynchophorus ferrugineus* Olivier (Coleoptera: Dryophthoridae), *Semanotus laurasii* Lucas (Coleoptera: Cerambycidae), and *Monochamus galloprovincialis* Olivier (Coleoptera: Cerambycidae), which have become serious threats to ornamental and productive trees all over the world such as palm trees, cypresses, and pines. Knowledge about their flight potential is very important for designing and applying measures targeted to reduce the negative effects from these pests. Studying the flight capability and behaviour of some insects is difficult due to their small size and the large area wherein they can fly, so we wondered how we could obtain information about their flight capabilities in a controlled environment. The answer came with the design of flight mills. Relevant data about the flight potential of these insects may be recorded and analysed by means of a flight mill. Once an insect is attached to the flight mill, it is able to fly in a circular direction without hitting walls or objects. By adding sensors to the flight mill, it is possible to record the number of revolutions and flight time. This paper presents a full description of a computer monitored flight mill. The description covers both the mechanical and the electronic parts in detail. The mill was designed to easily adapt to the anatomy of different insects and was successfully tested with individuals from three species *R. ferrugineus*, *S. laurasii*, and *M. galloprovincialis*.

## 1. Introduction

In the order of Coleoptera, there is a large number of species that constitute a serious danger to trees and plants, causing negative effects upon them during their feeding and reproduction. Reducing crop productivity or degrading the quality and good-looking appearance of trees and plants in gardens and landscapes are some of these effects. Moreover, in some cases, the negative effect is devastating, as it entails the death of the plant infested by such insect. Chemical or biological treatments have been developed to successfully fight against these kinds of insects. However, these treatments are not always completely effective. This is the case of insect borers that hide inside trees, making chemical and biological spraying difficult to reach them. For example, the Cerambycid beetle *Monochamus galloprovincialis* Olivier (Coleoptera: Cerambycidae) usually affects ill or even dead pine trees, but its real risk is that this insect is a vector for the nematode *Bursaphelenchus xylophilus* Steiner & Buhrer (Nematoda: Parasitaphelenchidae) which may kill a tree in just a few months.

Another example is *Semanotus laurasii* Lucas (Coleoptera: Cerambycidae) that directly attacks cypress trees, eventually producing the death of the plant. Furthermore, more than 20 years ago, *Rhynchophorus ferrugineus* Olivier (Coleoptera: Dryophthoridae) was introduced in Spain, and is today considered a global pest affecting different palm species on all continents [1]. Some important advances have been achieved, both in the knowledge about these insects and in the development of detection, prevention, and control mechanisms, but the pests are still out of control in several areas.

Knowledge about these insects, concerning all aspects of their biology and behaviour, is absolutely necessary if we are to find ways to defeat them. Researching aspects such as feeding habits, life span, host preferences, spread behaviour, and so on, involve several disciplines and collaborative efforts between researchers from different areas.

One of these factors is the flight skill of insects. Obtaining knowledge about parameters like maximum flight distance or specific flight patterns may help to develop strategies and procedures to prevent the spread of the pest such as pest outbreak management or proper trap locations [2]. Such studies may be accomplished indoors or outdoors.

In outdoor studies, the collected data is very close to reality, including environmental factors like wind and temperature. However, when insects are able to fly long distances, a huge effort is necessary to monitor their displacements. Several examples of outdoor study techniques exist. Traps are used in the technique of mark-release-recapture [3,4,5]. Recapture is avoided in [6] where insects are labelled with emitting radioisotopes that allow detecting them even if they are inside a tree. In recent years, radioisotopes were substituted by a radio-transmitter attached to the insect [7,8].

Indoor studies offer the possibility to obtain data about the potential behaviour of insect flight. Furthermore, they allow us to work with quarantine pests, because releasing these kinds of insects into the wild is not possible, like in the case of *R. ferrugineus*.

Another advantage of laboratory studies is the possibility to work with a large number of individuals; this may make the research easier and faster. However, the collected data must be carefully analysed before it can be translated to outdoor realities.

Indoor studies are usually performed by means of three techniques: static tethering, flight balances and pendulums, and flight mills [9].

In static tethered flight analysis, the insect is constrained in a fixed position, and flight behaviour is derived from the air displacement produced by wing movement [10] or by measuring the forces produced in the structure where the insect is attached [11].

In flight balances and pendulums [12], the insect is attached to the end of an arm, and the arm is attached to a pivot that allows it to ascend or descend as the result of insect wing beating. Pendulum-based monitoring presents a similar operation [13]. In both balances and pendulums, a scale is located behind the insect to measure the lift force produced.

The tethered flight mill method is considered a model system for laboratory analysis of insects flight behaviour [14], and has been successfully used to study the flight performance of a large number of species belonging to different orders.

However, the data and results obtained from the use of a mill must be accepted with some scepticism. The authors are convinced that the flight performance of an insect tethered to a mill is not the same as that obtained when an individual freely flies outdoors. Environmental parameters, like changes in daylight or the presence of sexual and feed attractants may affect the potential distance an insect is able to fly. A clear example is a tail wind helping the insect fly longer distances with less effort. Despite these constraints, knowledge coming from flight mills may be very useful for obtaining data about basic flight attributes [15]. Furthermore, it may also be useful for improving knowledge about the insect by other means, like outdoor experiments. In this case, data collected from the flight mill may help to arrange traps in a mark-release-recapture study, for example.

## 2. Rationale

There is a need for a device like a flight mill to carry out studies on insects that pose environmental threats or jeopardize agriculture. This mill needs to be adapted to the particular characteristics of such insects [15]. Therefore, it is important to show, with full detail, the design, construction, and use of this kind of device as a resource to help fight these pests.

This paper presents a detailed description of the construction, instrumentation, and use of a flight mill. The mill was designed with insects of different sizes and weights in mind, and one of its main goals is that the mill must easily adapt to a variety of insects, both in its hardware and software aspects. Furthermore, allowing for the reproduction of this design is an important goal.

The idea of tethering an insect to some structure and studying its flight is not new. In fact, it is several decades old. In 1952, Krogh and Weis-Fogh [16] presented a so-called roundabout for studying sustained flight of swarms of locusts, up to 32, using a stroboscope to measure the speed of the periphery and derive the flight intensity of each individual.

In [17], a flight mill was presented to allow semi-free flight of one individual of the Mexican fruit fly based on a vertical shaft with magnetic flotation. The insect is attached to an arm and the arm is attached to a magnet. This magnet is placed over another magnet facing like poles, therefore repelling it. To keep the floating magnet in place, a hypodermic syringe needle is inserted through both magnets, acting as an axle. Its authors claim null vertical friction, but some friction still remains between the axle and the floating magnet as it rotates that is reduced by covering part of the axle with Teflon.

In [18], the flight mill was built with magnetic bearings that vertically hold a steel entomological pin. A silica capillary builds the arm where individuals of leafhoppers (Hemiptera: Auchenorrhyncha) are attached. Magnetic floating or magnetic sustenance is commonly used to build flight mills [14,19,20].

However, the use of magnets presents some problems, mainly when the flight mill has to be adapted to different insects. In [21], the flight mill based on magnetic bearings was used to study the emerald ash borer (EAB), Agrilus planipennis Fairmaire. As stated in the aforementioned work, in the first experimental attempts, the larger size and greater strength of this beetle (as compared to leafhoppers) allowed them to wrest themselves free from the magnetic field after a few revolutions. More powerful magnets were then used, but that implied an increase of the torsional drag and the effort required to rotate the mill, possibly affecting the flight speed or its duration. Even more, some insects may be unable to start flying.

Thus, the strength of the magnetic field should be adjusted as a function of the insect being studied, and this tuning requires changing magnets, so significant modifications to the flight mill may be required in order to study different insects.

Some insects start to fly when tarsal contact with a substrate is removed [22]. For these kinds of insect, a mechanism to allow the insect to land may be necessary in the flight mill. This way, the individual may stop and restart flying at will, avoiding forcing the insect to fly until it becomes exhausted. With this goal in mind, [23] used a balance where an attached moth can take off and land freely. However, this balance allows measuring the time the moth is flying, but not the distance, an important parameter when studying pest insects.

The insects studied with the flight mill presented in this paper do not present tarsal reflex, and they start and resume flying freely even with no ground contact. During experimentation, some insects were observed with closed wings and moving legs like they were walking while hanging from the arm.

In order to build a flight mill with low friction to interfere as little as possible with the insects’ flight, and easily adaptable to insects of different sizes, the authors propose building a flight mill with ball bearings, similar to the one used in [24]. The advantage of the ball bearing is that it offers a robust union for its use with large size and mass insects like the red palm weevil. But at the same time, the friction of the ball bearing is very low, so it is also suitable for small, low strength insects. Regarding the arm, most previous works focus on reducing friction in the arm-base joint, but they pay little attention to the total mass the insect needs to move when flying. In this work, we use carbon fibre rods to build a light but steady arm.

Another important aspect when designing and building a flight mill is the way to instrument it. There are several options for detecting movement and counting revolutions. [25] use a miniature lamp and a phototransistor to detect the interruption of the light received by the phototransistor. [19] use a photoelectric switch to detect the passage of the arm. The same function is performed using an infrared emitter and receiver in [21]. The advantage of using infrared light is to avoid interference from visible light, so visible wavelength light can be manipulated according to the behavioural requirements of the insects being studied. In [24], a bike milometer is used to directly acquire statistical data about speed and distance. The milometer magnet is attached to the arm and the milometer sensor is attached to the mill pivot. Although some milometers may be connected to a computer and therefore record data automatically, there is no possibility of obtaining raw data about arm revolutions and only overall information is gathered.

Finally, arm revolution events, together with the time they occur, should be recorded in a computer system in order to automatically post-process these data and extract derivative magnitudes about the insect flight. Several works, including [12,14,25,26] employ hardware designed ad hoc to connect the arm revolution detector to a computer.

The flight mill described in this paper uses an infrared emitter and receiver to detect the movement of the arm when it interrupts the infrared beam, the same approach followed in other works like [21]. The output of the detector is connected to a standard PCI local bus [27] data acquisition card (DAQ) installed inside a personal computer where infrared beam interruptions are recorded.

This paper presents details about the connection of the infrared detector with the DAQ, sampling frequency, and developed software in order to help other research groups reproduce the design. Any similar DAQ may be used with no changes in the hardware and minor changes in the associated software.

Finally, the software architecture is presented. The data acquisition and processing is carried out by two separate applications. The first one records events in the mill without regard to the attached insect, but considering the mill’s mechanical characteristics, like the arm length and the number of infrared beam interruptions per revolution.

The second application processes the previous raw records, also taking into account insect-related parameters as described in the following sections. This second application provides a list of single flights including flight time and distance, and also a summary of the whole experiment.

Using two separate applications to obtain these results increases the amount of stored data, but it allows performing a single flight campaign, and carrying out several analyses afterwards, changing insect-related parameters without having the insects fly again.

## 3. The Flight Mill

**Figure 1:**
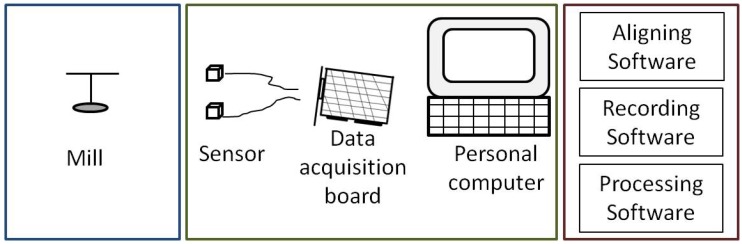
Functional blocks of the proposed flight mill.

The flight mill developed in this work may be divided into three main blocks, as shown in Figure 1. The first block is the mechanical mill where insects are attached and allowed to fly. The second block is the mill instrumentation for collecting information about mill rotations. This instrumentation is accomplished by means of an infrared sensor that detects arm revolutions. A computer with an off-the-self data acquisition board records the data coming from the sensor. The last block is a set of three software applications. The first is used to align the sensors. The second is devoted to interacting with the data acquisition card and storing raw data in the computer about arm rotations. And the third application processes the raw data according to user settings and produces results about several flight parameters, like distance and time flown, among others.

### 3.1. The Mill

**Figure 2:**
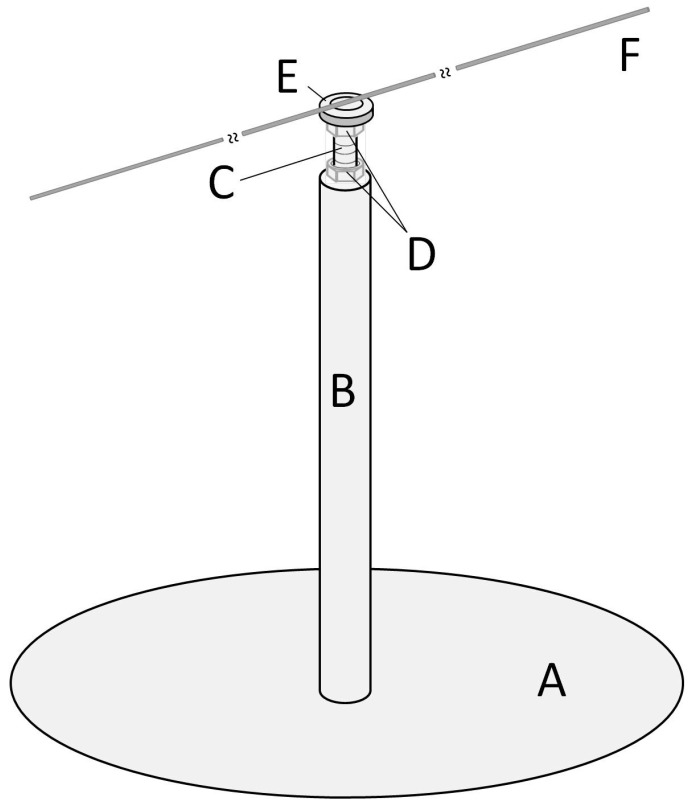
Mill design.

The mill is based on the design of [24] but incorporates some modifications. The main elements are the base, pivot, arm, and joint between the pivot and arm. Detailed descriptions of these elements and the way they are joined follows. Letters in brackets refers to elements in Figure 2.

A heavy iron disc (A) (⌀ = 200 mm, 3.7 mm thick and weighing close to 900 gr) is used as the mill base, and it has been chosen to provide stability. The bottom side of this disc is covered with foam (thickness = 2 mm) to reduce the effect of vibrations that may come from the facility where the mill is installed. At the centre of the base, an iron rod is attached vertically (B) (⌀ = 12.2 mm, length = 150 mm) acting as the mill pivot. The pivot is attached by welding. An endless screw is at the end of the pivot (C) (⌀ = 4 mm, length = 150 mm), and inserted and screwed in. The insertion depth is about 30 mm and may be secured by a nut (D) located at the lower end of the screw. A second nut (D), close to the upper end of the screw is used to adjust and secure a miniature flanged ball bearing (E). Both nuts are hex nuts compliant with standard DIN-934.

The miniature ball bearing (E) is the mill’s key mechanical component. Its purpose is to allow the mill arm to move with low friction and avoid any lever effect due to the insect weight. The ball bearing’s main parameters are: manufacturer Minebea Co., Tokyo, Japan; width = 3 mm; internal diameter = 4 mm; external diameter = 8 mm; flange diameter = 9.2 mm. To this ball bearing, a washer (E) (internal ⌀ range = [8 mm to 8.4 mm], external ⌀ range = [14 mm to 15 mm]) is attached. Cyanoacrylate glue (Super Glue-3, Henkel Ibérica, Barcelona, Spain) is used to attach the washer to the ball bearing flange. Washer dimensions are critical because avoiding contact between it and the inner ring edge is mandatory, and at the same time the washer must offer the largest possible surface for attaching the mill arm. The weight of the union ball bearing and washer (E) is 2.04 gr.

**Figure 3:**
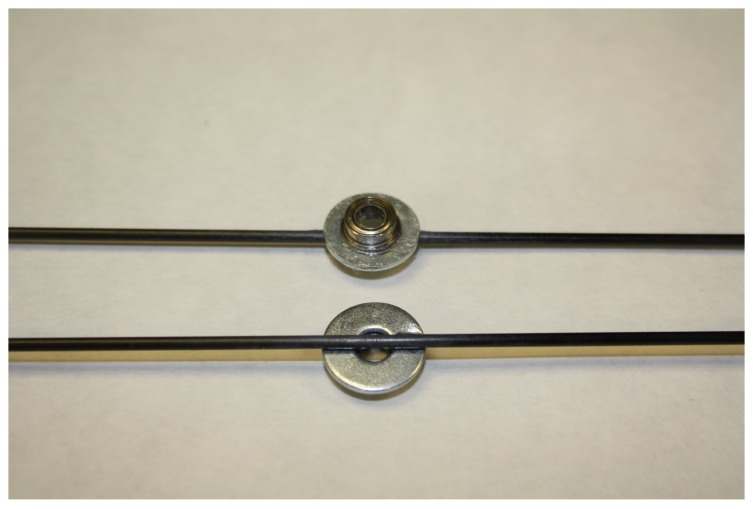
Detail of the union of the fibre carbon rod, washer, and ball bearing.

Two options were used to build the mill arm for adapting it to different insect species. In both cases, the arm is a carbon fibre rod (F) (Eolo Sports Industrias, Gijón, Spain). For large insects, such as *R. ferrugineus*, the rod is 2 mm in diameter, 640 mm in length, and weighs 3 gr. For smaller insects, such as *S. laurasii*, the rod is 1 mm in diameter, 480 mm in length, and weighs 0.54 gr. The fibre rod attaches to the washer using hot silicone (Salki, Comersim S.A.U., Pamplona, Spain) as shown in Figure 3. One revolution of the thicker arm provides for a flight path of 2.01 m and 1.50 m for the thinner rod. Special care should be taken to join the arm to the ball bearing-washer system, centring the arm both lengthways and laterally to the rotating axis of the ball bearing to avoid deviation in the flight path length.

**Figure 4:**
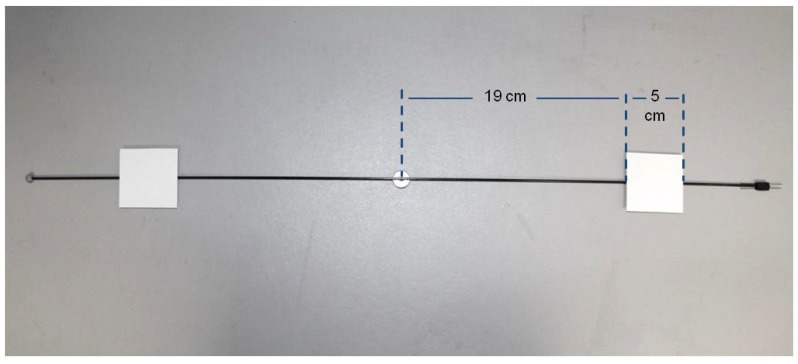
Detail of card stock, counterweight, and insect attachment pins.

At one end of the arm, a plasticine counterweight (Jovi, S.A., Barcelona, Spain) is placed to compensate for the weight of the insect. At the other end of the arm, there are two parallel, horizontal directed pins for attaching the insect (see Figure 4).

**Figure 5:**
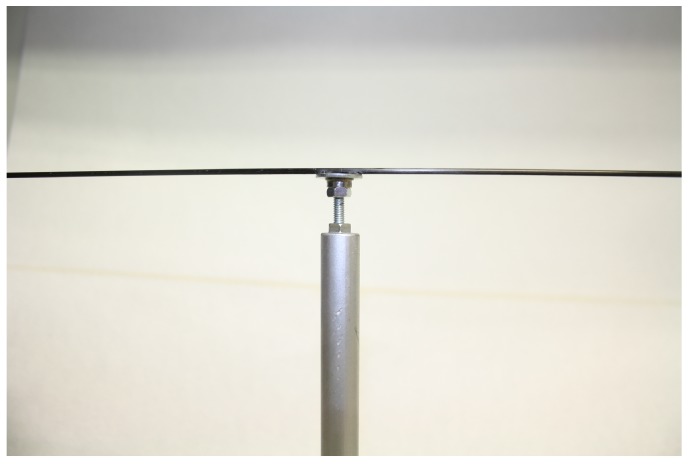
Arm assembly in the endless screw supported by the upper nut.

**Figure 6:**
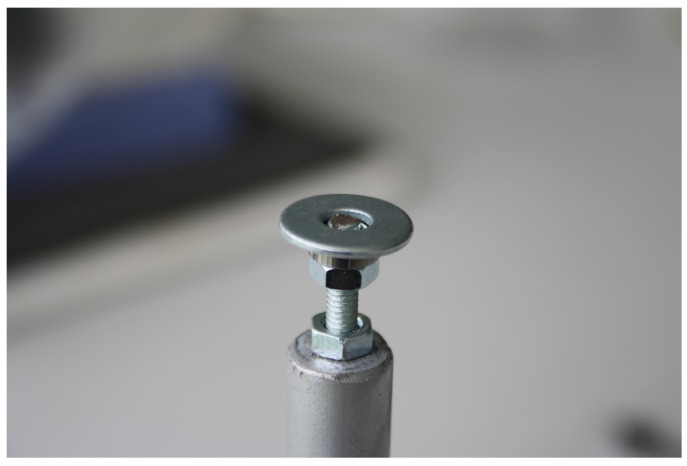
Detail of the washer-ball bearing assembly supported by the nut, without the arm.

**Figure 7:**
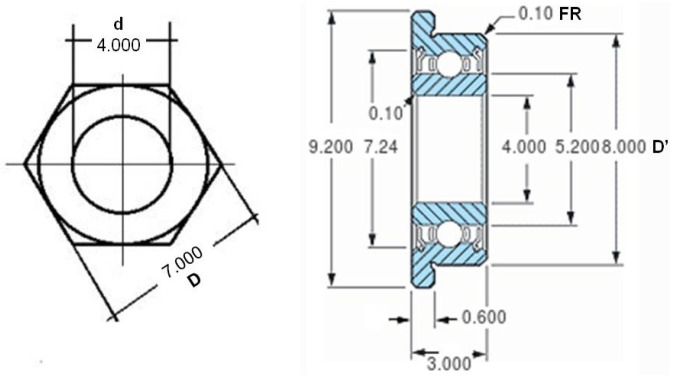
Dimensions of DIN-934 hex nut and ball bearing from Minebea Co., Tokyo, Japan.

Finally, the mill is assembled inserting the arm-washer-ball bearing (see Figure 5 and detail in Figure 6) at the top of the screw. For the assembly, the upper hex nut has been placed 3 mm from the top end of the screw. Then the ball bearing rests at the end of the screw, supported by the nut. In Figure 7, the dimensions of a DIN-934 hex nut and the ball bearing described previously are shown. For a nut with hole diameter (d) of 4 mm, the flat diameter (D) is 7 mm, the same as the inner diameter of the outer ring of the ball bearing outside diameter (D’) minus the fillet radius (FR). This way, contact between the inner and outer rings of the ball bearing is avoided, and the arm can move freely, with only the very low friction of the ball bearing.

**Figure 8:**
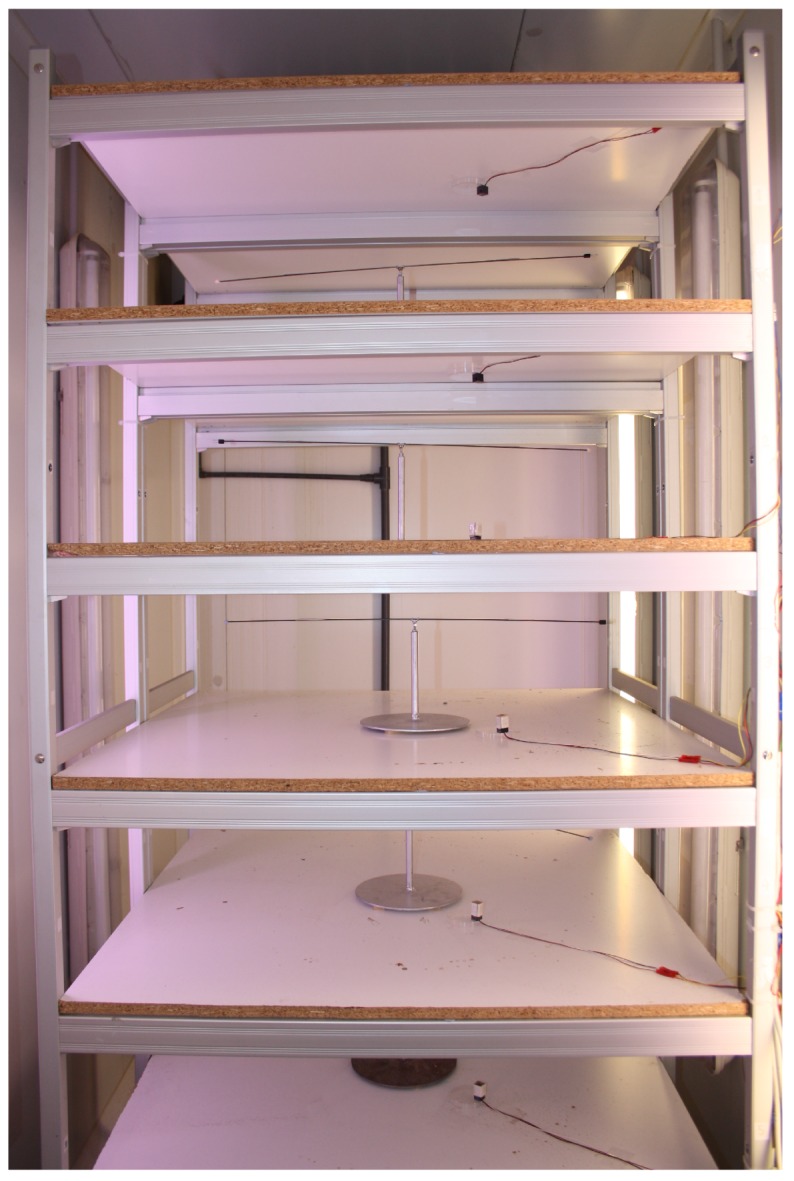
Rack composed of 5 flight mills.

**Figure 9:**
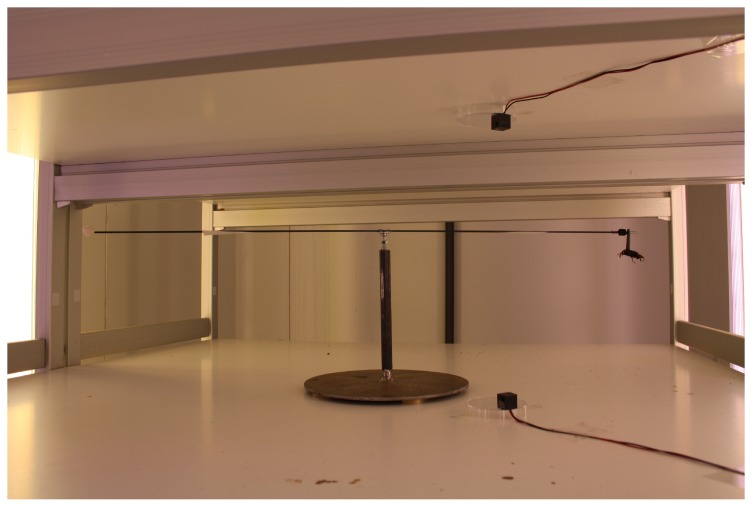
Rack detail with an *R. ferrugineus* individual tethered to the mill.

The mill is installed on an open rack, composed of six chipboard shelves (length = 90 cm, width = 90 cm, thickness = 2 cm) with a space of 30 cm between shelves. A total of five mills have been installed in this rack, shown in Figure 8 and Figure 9.

### 3.2. Instrumenting the Mill

The flight mill’s main objective is to collect data about the time and distance the attached insect is able to fly. Derivative magnitudes like speed, average flight distance, and maximum flight time are of interest also. Flight distance may be measured by simply counting the number of arm revolutions. But measuring the time is needed in order to obtain the remaining magnitudes.

We define an experiment as the process of attaching an insect to the mill, allowing it to fly for a predefined time (normally hours), and then removing it from the mill. To obtain the results mentioned before, taking timestamps of start and end flying events is not enough, since the insect may take breaks or perform irregular flight periods. In order to obtain accurate data about flight behaviour, accurately measuring the time of each arm revolution while the insect is attached to it is necessary.

**Figure 10:**
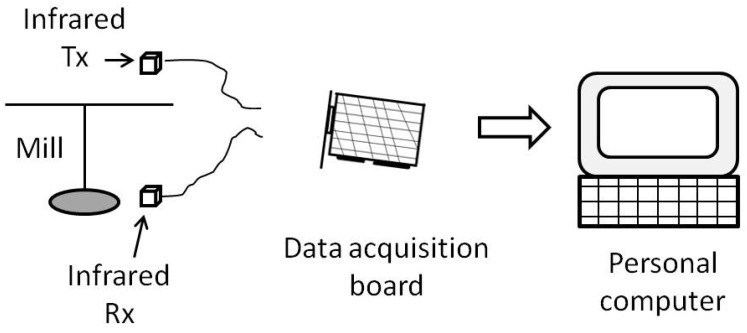
Block diagram of mill instrumentation.

To measure the time the arm takes to make a revolution, some electronic devices are used: first, an infrared emitter and receiver are coupled to produce and detect an infrared beam that is interrupted by the rotating arm; second, a data acquisition board with digital inputs collects the information from the infrared devices; and third, a personal computer populated with the data acquisition board and running Microsoft Windows XP stores and performs calculations on the collected data. Figure 10 shows a block diagram of this arrangement.

The infrared emitter and receiver are used to detect the mill’s arm passing. The receiver is located at the base of the mill, close to an edge. It is attached to the shelf with hot silicone. The emitter is attached to the bottom of the shelf above it and is also attached with hot silicone. Here are some considerations about the use of the infrared detector:

- The infrared beam produced is not very wide, about 6 mm in diameter, so the emitter and receiver alignment is important. A simple computer application, described later, is used to help the user align the beam.
- The receiver’s sensing area has a 5.59 mm diameter. Taking into account the width of the mill’s arm (1 or 2 mm), it may be possible that the arm is not wide enough to completely block the beam from the infrared detector. To ensure the beam is completely interrupted when the arm crosses it, a small (50 mm × 50 mm) rectangular piece of card stock is added to each arm leg 19 cm from the centre of the arm (see Figure 4).
- By adding card stock to the mill arm, we can guarantee that the infrared receiver has enough time to detect the beam interruption. Using some simple geometry calculus, it can be determined the beam is interrupted for 3.6 ms considering the insect is attached 24 cm (radius of the short fibre rod) from the centre of the arm, and assuming the insect flies at 50 Km/h. The lower the flight speed or the longer the arm, the longer the time the beam is interrupted and thus the detection is easier for the infrared receiver. Each complete revolution of the mill arm blocks the infrared beam twice, so actually half revolutions are counted.

Both the infrared emitter and receiver are connected to a data acquisition board (APCI-IB40; ARCOM CONTROL SYSTEMS, Cambridge, UK). This board allows the connection of up to 40 digital inputs/outputs (DIO), and is also able to power low consumption devices using a 5 volts output and a GND terminal. The power consumption of each emitter and receiver pair is about 10 mA; as the rack allows for the installation of five mills, the total power requirement for the installation is about 50 mA. This is low enough to directly power all five pairs of infrared emitters and receivers from the acquisition board.

**Figure 11:**
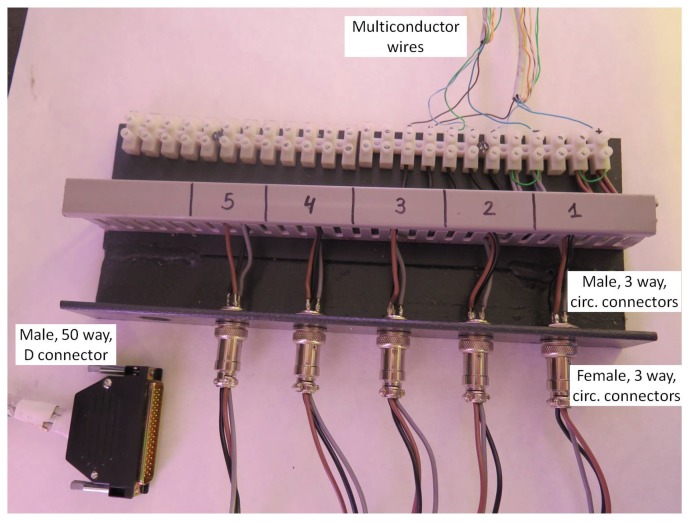
Detail of interfacing data acquisition board and infrared emitters and receivers.

Five DIO from the board are configured as inputs, and the output of the infrared receivers are connected to them. The APCI-IB40 board has an outer, female, 50 way, D-type connector, where digital inputs and outputs can be connected. Also, from this connector, a DC power supply of 12 V and 5 V, as well as ground, can be obtained. Direct wire plugging to this connector is not possible, so a male, aerial, 50 way, D-type connector with two unshielded multi-conductor cables (Alphawire; Sunbury-on-Thames, UK) was used. One end of the ribbon cable was soldered to terminals of the aerial D-type connector and the other end was stripped and screwed to a rack with terminal blocks (Sofamel; Barcelona, Spain). Terminal blocks were connected to a male, panel mounting, 3-way circular connector. Single wires were used to connect infrared emitter and receiver terminals to a female, 3-way aerial circular connector (Amphenol; Wallingford, CT, USA). This solution provides easy facility maintenance. Detail is shown in Figure 11.

The APCI-IB40 board also includes four 16-bit timer/counters nominated timer/counter 0 to timer/counter 3. Timer/counter 0 is used to trigger an interrupt signal at a fixed frequency based on a programmable divider from its clock source, which is selectable to be 1 MHz, 100 KHz, or 10 KHz. In order to not miss any activation of the five outputs coming from the infrared receivers, these signals must be sampled using a period shorter than the minimum beam disruption time, which in turn depends on the insect’s speed. This way, timer/counter 0 is configured to raise an interruption every 5 ms, allowing measuring arm linear speeds up to 35 Km/h. To the authors’ knowledge, this speed is greater than the maximum speed of any insect under study [28]. An interrupt is raised every 5 ms configuring timer/counter 0 in auto-reload mode, with a reload value of 500 counts and input clock frequency of 100 KHz.

### 3.3. Software

Three applications were developed to set up the system, to record raw flight information, and to process the recorded information. The partition of the software into three applications aims to improve the flexibility of the system. All three applications are programmed in the C++ language [29] using Borland C++ Builder version 6.0 [29].

#### 3.3.1. Infrared Beam Alignment

**Figure 12:**
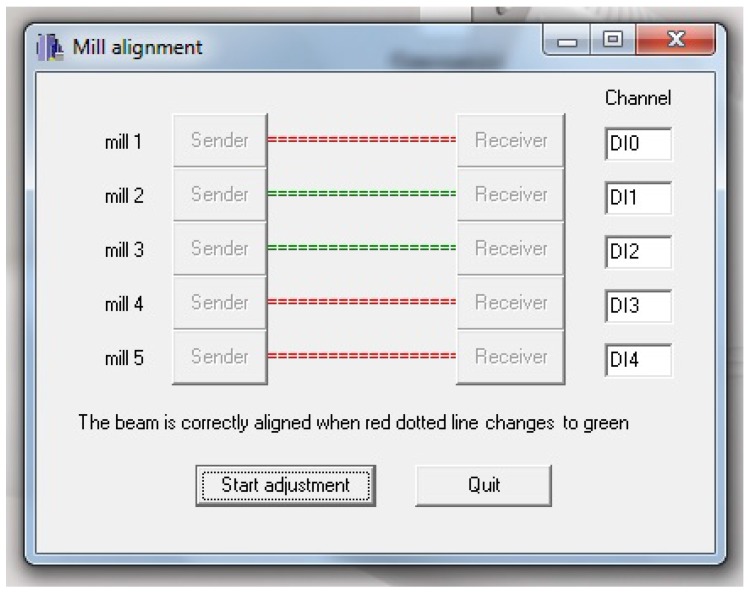
Interface of application for infrared beam alignment.

A simple graphical application is used to help the user in the infrared beam alignment procedure. The application configures the APCI-IB40 board to generate an interrupt every 5 ms by means of the timer/counter 0. The interrupt service routine checks the output of the five infrared receivers and shows a coloured label indicating whether the receiver is detecting the beam (green label) or not (red label). A human operator may then adjust the receiver position until the label changes to green, indicating the emitter and receiver are aligned. Figure 12 shows a capture of the graphical interface of this application where only the infrared beams of mills 2 and 3 are correctly aligned.

#### 3.3.2. Capturing Events

**Figure 13:**
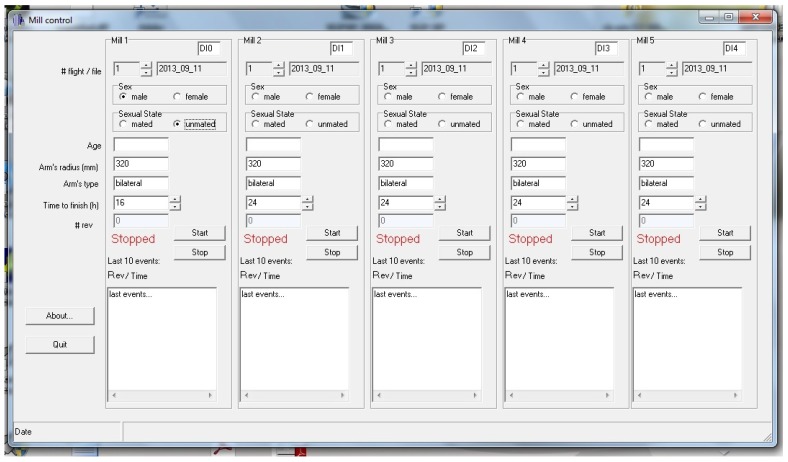
Interface of the application for capturing events (arm revolutions).

This application is in charge of the detection of events originating from the five mills in the installation and recording the associated information into a file in the computer hard disk drive. The graphical interface of this application (see Figure 13) allows the user to customise several experiment parameters. What follows is the list of per-mill parameters used:

- Digital channel number of the acquisition board where the infrared receiver signal is connected.
- Number and name of the file where events were recorded. An automatic filename is provided by the application using the mill number, the experiment number, and the experiment date. The experiment number is automatically increased when the user ends an experiment. In all cases, the user may change the file name and choose a filename that best suits their needs.
- Sex, age, and sexual state of the insect.
- Arm length. This option provides flexibility to the mill and allows using different arms depending upon the insect under study.
- Arm type, indicating if the arm interrupts the infrared beam once or twice per revolution. This option is intended to deal with other ways of detecting the arm movement.
- Desired experiment time limit. Once this time is reached, the application stops recording events.

All these parameters work individually for each of the five mills.

The application offers two buttons for each mill, one to start capturing events and the other to manually stop the capture. When the start button is pressed, a new file is created and experiment parameters are stored in it. A simple plain text format is used to arrange the data inside the file. Also, the current date and experiment duration is written in the file.

The application configures the timer/counter 0 in the acquisition board to generate an interrupt every 5 ms. This configuration is accomplished only once, the first time the user presses one of the five start buttons. Then, every 5 ms, the interrupt service routine checks the state of the infrared sensor for the active mills (those in which their start button has been pressed but neither of their stop buttons has been pressed nor the experiment time limit has been reached). Because the time the beam is interrupted by the card stock piece attached to each arm leg depends on the insect’s flight speed, the time the infrared beam is broken may last quite longer than 5 ms, so simply checking the infrared receiver output does not work: a single beam interruption produced by a slow insect may be detected several times and lead to a false larger number of counts. To avoid this problem, the interrupt service routine detects the falling edge (a state change from a high to low logic level from two consecutive samples) in the receiver output using a Boolean variable to store the state of the receiver output in the previous sample and comparing it with the current receiver output state. Every falling edge is recorded in the file, coupled with the current system time (hours, minutes, seconds, and milliseconds provided by the operating system).

The interrupt service routine performs two more operations. First, it checks if any experiment has reached its time limit (the user-selected experiment time) in order to stop checking the sensor output, close the file of events, and increase the experiment number for the related mill; and second, for mills with a new event, updates the application graphical interface with some information about the recorded events: the number of times the beam has been disrupted and the list of the last ten events with their associated timestamps.

When the user presses the stop button or the experiment time limit is reached, the file containing all the information for the experiment is closed and the experiment number for the stopped mill is automatically increased. Then the graphical interface shows the mill is ready for a new experiment. From this moment forward, a new experiment may be configured and manually started.

#### 3.3.3. Processing Recorded Files

This application is dedicated to process the data recorded by the *Capturing events* application described in the previous subsection. This last application is a purely software one and does not interact with the mill hardware. It would be easy to include all the required functionality in a single application, but the authors preferred to create separate applications because some parameters and options of this application depend on the insect under study, so more and frequent modifications are expected here than for the application that records raw events, an application that relies on hardware devices that are not easily modifiable. Even when the arm is changed to adapt it to a different kind of insect, all the program functions remain the same, and only some parameters, such as the length of the arm, need to be changed in the application devoted to recording events.

Having a separate application to process the raw recorded data is also advantageous in the sense the same raw data may be used to perform multiple different analyses with modified parameters without the need for insects to fly again. From a naive point of view, counting the half revolutions of the arm is enough to determine the distance the insect has flown for the whole experiment, and using the start and end time is enough to calculate the average flight speed. But there are several reasons for performing a detailed analysis of the recorded data:

- While flying for several hours, the insects may take several breaks, so stops and pauses have to be detected.
- When an insect stops flying, a backward movement may happen while the arm is near the infrared detection area, producing two breaks of the infrared beam with a very short in-between interval that leads to an erroneous flight speed calculation if this possibility is not taken into account
- When an insect needs or wants to stop flying, it is used to landing and stopping. However, while attached to the mill, an insect may stop flying but remain in movement until friction gently slows and stops it. Counting the revolutions the arm completes after the insect closes its wings leads to an overestimation of the distance flown.

The authors propose the use of single flights, like proposed in [30], to address the aforementioned issues. For the duration of an experiment, which may span from a few minutes to several hours to even longer than a day, the insect behaviour is divided into single flights. A single flight is defined as the period between two consecutive stops, and a stop is defined as a time period without arm revolutions or with significantly slow revolutions.

**Figure 14:**
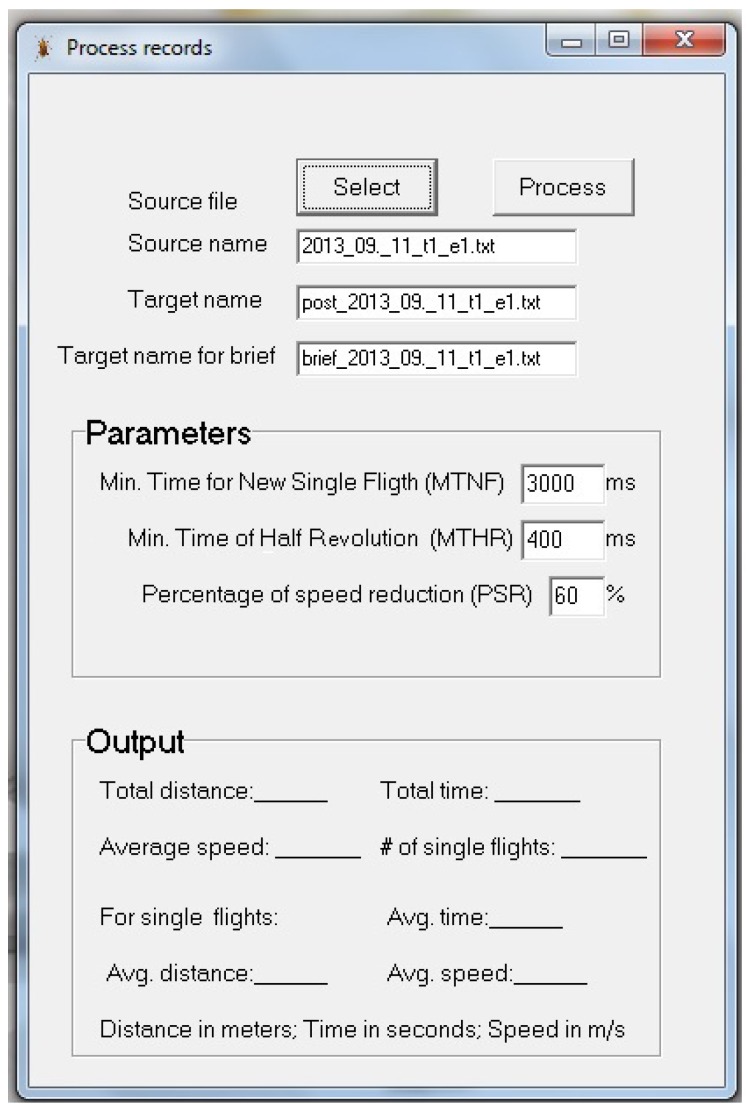
Interface of the application for processing events.

Stop periods are determined after an experiment has finished as a pre-processing step before the analysis of captured data. The stop period durations are specific for each kind of insect. As the file with the recorded raw events is always available, the researcher can change the duration of this period and re-process the recorded data, gathering information about the insect’s flight behaviour. The application presents a graphical interface (see Figure 14) where the user can set three parameters to define when a single flight starts and stops. The possibility of changing these parameters allows working with different insects. Moreover, the division in two separate applications, one for recording hardware events and the other for explaining them, enhances the flexibility and adaptability of this proposal. Details of the parameter follow:

- Minimum time to start a new single flight (MTNF). If the time between two arm half revolutions is longer than this parameter (MTNF), a new single flight is considered. That is, it is assumed the insect stopped and rested for a while, then started a new single flight.
- Minimum time of a half revolution (MTHR). If the time between two consecutive beam interrupts (*i.e.*, an arm half revolution) is shorter than this value, it is considered a backward movement or simply as an erroneous detection, because it means that the flying speed of the insect is too high. In this case, the second beam break is discarded and the next raw event is considered.
- Percentage of speed reduction (PSR). When the insect stops flying, there is still movement, but its speed gently decreases until stopping completely. The flight speed is more or less constant during a single flight, so a significant speed reduction may indicate that the insect has closed its wings and is moving only due to system inertia. When the average speed of the actual flight is reduced in the percentage indicated, the subsequent arm revolutions are discarded until a break in the flight or a significant increase of speed occurs. The meaning of the latter is that the insect has rested for a brief time, and so we consider the current single flight is still valid.

The graphical interface allows the user to select the file with recorded raw events as the source of the analysis, and it creates a new file adding the *post* prefix to the original name as the target file. In this file, the results of the whole experiment and single flights are stored using plain text. The information stored in the target file is described below:

- Source file name.
- All the data included in the source file related to experiment characteristics, like the age, sexual state, and sex of the insect, arm characteristics, and those previously described.
- Total distance flown by the insect as the cumulative sum of the distance of single flights.
- Total time flown by the insect as the cumulative sum of the duration of single flights.
- Average speed for the whole experiment.
- Number of single flights.
- Average distance for single flights.
- Average duration for single flights.
- Average speed for single flights.
- List of single flights, including for each flight, the following data: start and end time, number of revolutions (discarding those faster than the MTHR), elapsed time, distance flown, and average speed. This list is stored in plain text using a comma-separated values (csv) format [31,32] allowing the researcher to easily export this information to a statistical analysis application.

A second file is also created, adding the prefix *brief* to the source file name that contains the raw recorded events. In this file, only a brief summary of the results from the event analysis is stored in columns and the csv format. The purpose of this summary is to allow the automatic concatenation of lots of event-processed results to import all the information together into a statistical tool. The information is the same as in the full report except for the list of single flights. Each field in this file is stored in a column without headers. Concatenation may be easily done by means of batch scripts using operating system console commands.

## 4. Experimental Section

Experiments presented in this paper were devoted to test the proper mill operation studying different insects, and to collect insect flight data.

First of all, special calibration is not needed since there is neither analogue-digital conversion nor specific measurements capable of being calibrated during the mill operation. There are only two variables measured in this work. On the one hand, we measure the distance covered by the insect in the mill. This distance is derived from the radius of the arm (a fixed and known value measured with a precision ruler) and the number of revolutions completed by the insect. The second variable is the elapsed time between two consecutive interrupts of the infrared beam (a distance of a half revolution). To take the corresponding time measurements, we used the PC clock timing system that is precise enough (order of microseconds) to accurately measure the half revolutions elapsing time of the insect flight. Also, the proper functioning of the overall system has been manually verified by acting on the mill to generate all possible events, contrasting the information recorded by the system with the one observed by an operator with a stopwatch.

Several adults of three coleopteran borer species, *R. ferrugineus, S. laurasii*, and *M. galloprovincialis* were used.

A total of 163 *R. ferrugineus* unmated adults (86 males and 77 females) were used for the experiments, obtained from cocoons collected from infested *P. canariensis* palms in the town of Sueca, in eastern Spain (latitude N $39^{\circ }\;12^{\prime }$; longitude W $00^{\circ }\;18^{\prime }$), between January and December 2012. The cocoons were held in individual sterilized 100 ml plastic containers with perforated lids and maintained in a climatic chamber at 25 ± 2 ${}^{\circ }$C and 65%±5% relative humidity. Adult emergence was checked once a day to determine their exact age and sex, after which the newly emerged weevils were returned to the containers. A piece of apple, replaced twice a week, was provided as a food source [33] until the insects were used in the tests.

*S. laurasii* adults were obtained from *Cupressocyparis leylandii* (A. B. Jacks. & Dallim.) Dallim. (Pinales: Cupressaceae) wood logs coming from gardens in the city of Valencia (latitude N $39^{\circ }\;28^{\prime }$; longitude W $00^{\circ }\;22^{\prime }$) in eastern Spain.

In the case of *M. galloprovincialis*, the adults were recovered from *Pinus halepensis* Mill. (Pinales: Pinaceae) wood logs coming from the 2013 fire in Cortes de Pallás (latitude N $39^{\circ }\;14^{\prime }$; longitude N $00^{\circ }\;56^{\prime }$) in the Valencia region of Spain. The wood logs for both species were kept in a climatic chamber under temperatures of 25 ± 2 ${}^{\circ }$C and 65%±5% relative humidity. Adult emergence was checked once a day to determine their exact age and sex, and the emerged adults were kept in individualized containers. Table 1 shows the number of insects collected.

Each individual was weighed with a precision scale (Acculab; ALC-210.4, Bradford, PA, USA) and the length of its body was measured longitudinally with a digital calliper (Comecta Corp.; Barcelona, Spain). Table 2 shows the main parameters of the insects used in experiments.

**Figure 15:**
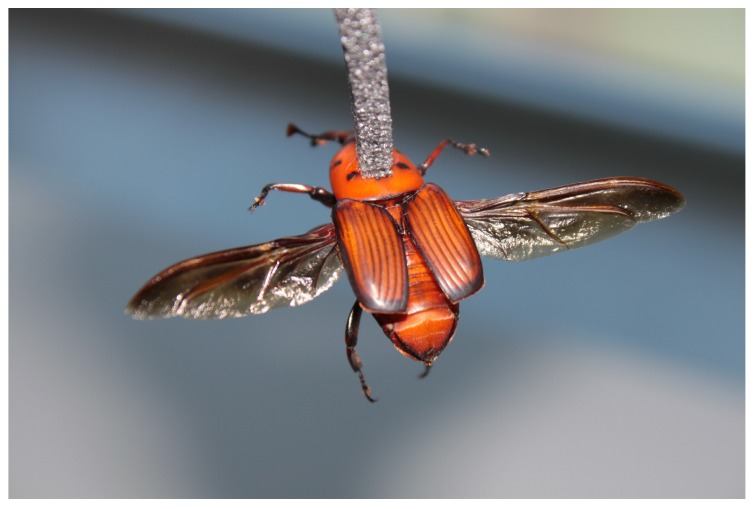
Detail of the tethering of an *R. ferrugineus* adult to a piece of foam.

For each weevil, a length of polyethylene foam (30x4x4 mm) is attached to its pronotum using cyanoacrylate glue (Super Glue-3, Henkel Ibérica; Barcelona, Spain) like shown in Figure 15. Then, the foam is fixed to two pins installed at the end of the arm. Five flight mills were simultaneously used in a climatic chamber, maintained at 25 ± 2 ${}^{\circ }$ C, 65%±5% RH, and constantly lit by non-flickering 58 W fluorescent (Philips Ibérica; Madrid, Spain) and Grolux lamps (Osram Sylvania Inc.; Danvers, MA, USA).

Because of the small size of *S. laurasii*, the arm for this insect was built with a carbon fibre rod 1 mm in diameter in order to reduce the mass the insect had to move, as described in Section 3.1. The arm structure, which includes the ball bearing, the washer, the arm, the two pieces of card stock, the foam, and pins used to attach the insects and the glue to keep all the parts joined, presented a total weight of 4.43 g. For the other two species, *R. ferrugineus* and *M. galloprovincialis*, the arm structure was built with a carbon fibre rod 2 mm in diameter, also described previously. The arm structure using the thicker rod presents a total weight of 6.90 g, including the same parts as the piece used for *S. laurasii*.

It is important to remark that changing the arm structure does not require any tools, since all parts remain joined together and the ball bearing rests freely on the vertical screw, stopped by the upper nut, and it can be easily removed and inserted.

Usually, these kinds of flying insects do not present tarsal reflex, so they do not start flying immediately after being left in the mill. This way, insects were left in the mill for 12 h, while the computer system monitored and recorded arm revolutions. After this period, computer records were observed to check that insects where able to fly when fixed to the arm. For *R. ferrugineus* and *M. galloprovincialis*, around 70% the individuals were able to fly (see Table 3). This percentage is similar to the data presented for other species [34]. For *S. laurasii* this percentage reduced to 22%. Further experiments would be needed to asses if this low number of flying individuals is because of the small size of the insect compared to the arm, or if it is an intrinsic property of this species.

In all cases, there was no relationship between the insects that didn’t fly and the mill they were attached to; several insects flew in all five mills. Recorded data was processed using the application described in Section 3.3.3. Different values for the minimum time to start a new single flight (MTNF), the minimum time of a half revolution (MTHR), and the percentage of speed reduction (PSR) were set for each kind of insect (see Table 4).

Table 5, Table 6 and Table 7 show the summary of the main flight parameters for *R. ferrugineus*, *S. laurasii*, and *M. galloprovincialis*, respectively. Parameters shown are number of single flights (NOF), total distance flown (TDF), longest single flight (LSF), flight duration (FD), average speed (AS), and maximum speed (MAXS).

In order to validate the results obtained above, we have checked them with respect other studies about the *R. ferrugineus* (RPW) flight potential. In particular, we have found a couple of works in the literature: an outdoor study in [3], and recently in [15] an indoor study of RPW flight potential by means of a flight mill. We compared the results provided by [15] with the ones obtained with our flight mill. Unfortunately, the experimental setup of both works were not the same, so we have to take care when comparing their results. The main differences between both experimental works (apart of the flight mills) are the following:

(a) The RPW individuals used in our flight mill came from cocoons, so they have no previous flight experience. However, in [15] the RPW individuals have been captured from traps, being all of them adults with proved flight capacity.
(b) The age of individuals. In our experiments all the individuals are less than three weeks old, with and average age of 8.25 days. The age of individuals used in [15] is unknown, but it is expected to be higher than the ones used in our experiments.
(c) The individuals size. As with individuals age, there are also differences in the size and morphology of RPW individuals depending if they come from cocoons or traps. The average weight and length of the individuals used in our study was 1.11 grams, and 32.83 mm, respectively. Whereas in [15], the average weight and length was 1.32 grams and 26.36 mm. So, our insects are longer and lighter that the ones used in [15].
(d) In [15], each flight test lasted 24 h per individual, with an average temperature of 27 ± 2 ${}^{\circ }$C. In our experiments the flight monitoring period was just the half (12 h) and the average temperature was 25 ± 2 ${}^{\circ }$C.

**Figure 16:**
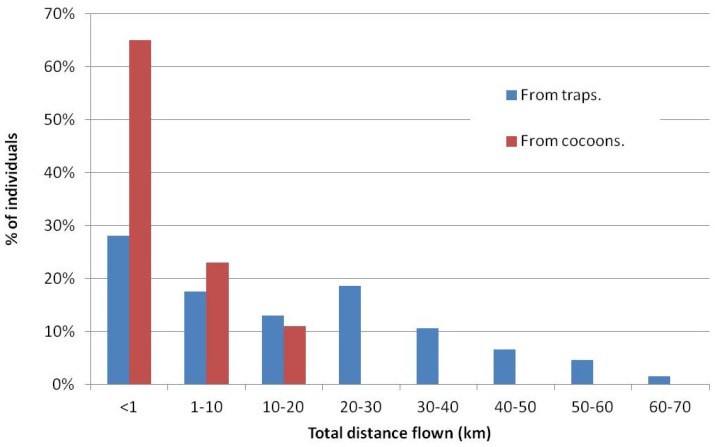
Total distance flown (in km) by individuals coming from traps [15] *versus* distance flown by individuals coming from cocoons (this work).

In Figure 16, we show the summary of maximum distances obtained in the experiments of Hoddle’s work [15] and the ones performed with our flight mill proposal. Taking into account the differences in the experimental setups, specially the differences in the flight test period and the individuals flight experience, we may observe that our tests show a significant high number (65%) of individuals that flew less than 1 Km when compared with the results found at [15]. However, the percentage of individuals flying from 1 to 20 Km is more or less equivalent in both studies. Our insects were not able to fly more than 20 Km in the 12 h flight test period.

The outdoor study described in [3] was able to estimate the flight potential of RPW individuals by means of mark, release and recapture techniques. The obtained results show that in average the individuals were able to fly between 1 and 7 Km in the lapse of 3 to 5 days.

Although there are differences between all the experiments we have analysed, we can conclude that (a) the results obtained in each of them are compatible; (b) their differences are mainly due to the experimental setups followed in each work; and (c) our flight mill design is able to measure the flight potential of RPW individuals.

## 5. Conclusions

This work presents a detailed description of a flight mill and specialized software applications dedicated to collecting and analysing flight data in order to study the flight behaviour of different coleopteran species. Differences in size and weight of the species promote the design of a mill with a hardware structure easy to adapt to different species. Also, the software developed to process and analyse the collected data is able to adapt to differences among the species under study.

Regarding the mill hardware, the relationship between the insect masses and the mill arm that the insect must move during its flight is of utmost importance. The mill has to be stable and able to keep the insect flying a consistent path, so some mass and rigidity is needed, minimizing at the same time the mass of the arm to mimic the conditions of free flight as much as possible. To achieve both requirements, the moving parts of the mill have been reduced to a carbon fibre bar, a washer, and the outer ring of a miniature ball-bearing. Movement detection required the addition of two pieces of card stock, increasing the mass.

The moving parts are joined together in a unique piece than can be easily changed without the need for tools. This allows easily working with smaller or larger insects, adapting the mass of the arm to the physical characteristics of the insect. Two arms were designed and tested: one piece with a 1 mm fibre rod and a total weight of 4.43 g including all parts; the other one with a 2 mm fibre rod and a total weight of 6.90 g, including all parts.

The experiments conducted show that both the mill hardware and its software perform properly. We have also compared the results obtained with our flight mill with the ones from [3,15]. The comparative study shows that the results provided by our proposal are compatible with the ones shown in the other works.

Future work is directed to improve the hardware to reduce the mass of the moving parts, mainly affecting the washer and the card stock parts. Using lighter materials or another way to detect movement may result in a 20%–40% reduction in mass.

## Figures and Tables

**Table 1:** Number of individuals collected for each insect species.

Species	Total	♂	♀
*R. ferrugineus*	163	86	77
*S. laurasii*	170	85	85
*M. galloprovincialis*	43	19	24

**Table 2:** Main morphological characteristics, body weight and body length, for males and females of the different tested species.

Species	Avg Weight ± SE (g)	Min Weight (g)	Max Weight Max (g)	Avg. Length ± SE (mm)	Min Length (mm)	Max Length (mm)
*R. ferrugineus* ♂	1.02 ± 0.020	0.53	1.62	31.08 ± 0.25	24	38
*R. ferrugineus* ♀	1.19 ± 0.030	0.51	1.60	34.16 ± 0.29	26	39
*R. ferrugineus* ♂ + ♀	1.11 ± 0.020	0.51	1.60	32.83 ± 0.27	24	39
*S. laurasii* ♂	0.16 ± 0.003	0.10	0.24	17.05 ± 0.25	11.87	22.34
*S. laurasii* ♀	0.20 ± 0.060	0.11	0.33	18.75 ± 0.31	11.20	24.69
*S. laurasii* ♂ + ♀	0.18 ± 0.004	0.10	0.24	17.90 ± 0.21	11.20	22.34
*M. galloprovincialis* ♂	0.42 ± 0.030	0.23	0.65	22.00 ± 0.47	18	25
*M. galloprovincialis* ♀	0.34 ± 0.020	0.22	0.54	18.90 ± 0.27	18	24
*M. galloprovincialis* ♂ + ♀	0.38 ± 0.020	0.22	0.54	20.20 ± 0.28	18	24

**Table 3:** Number of tested insects and the number of individuals able to fly from each insect species.

Species	Total Tested	Total Flown	♂	♀
*R. ferrugineus*	163	110	58	52
*S. laurasii*	170	39	22	17
*M. galloprovincialis*	43	29	15	14

**Table 4:** Parameters used for processing recorded events.

Species	MTNF (ms)	MTHR (ms)	PSR (%)
*R. ferrugineus*	2000	400	70
*S. laurasii*	3500	400	70
*M. galloprovincialis*	3500	400	70

**Table 5:** Summary of flight parameters for *R. ferrugineus* adults.

Parameters	Total (*n* = 110)			Males (*n* = 58)			Females (*n* = 52)		
	Mean ± SE	Max	Min	Mean ± SE	Max	Min	Mean ± SE	Max	Min
NOF	15.3 ± 2.8	233	1	20.1 ± 5	233	1	9.9 ± 1.5	59	1
TDF (m)	2780.2 ± 432.3	19,659.6	4	3309.2 ± 625	19,659.6	4	2190.1 ± 587.5	17,398.6	25.1
LSF (m)	1335.7 ± 229.9	11,244.9	4	1613.3 ± 317.3	9434.9	4	1026.1 ± 331.7	11,244.9	9
FD (min)	32.42 ± 5	260.7	0.1	39.1 ± 7.12	203.1	0.1	24.9 ± 6.9	260.6	0.4
AS (km/h)	3.7 ± 0.1	7.1	2.4	3.8 ± 0.1	7	2.4	4 ± 0.4	21	2.4
MAXS (km/h)	6.1 ± 0.1	9.3	2.9	6 ± 0.2	8.3	2.9	6.1 ± 0.2	9.3	3.4

**Table 6:** Summary of flight parameters for *S. laurasii* adults.

Parameters	Total (*n* = 39)			Males (*n* = 22)			Females (*n* = 17)		
	Mean ± SE	Max	Min	Mean ± SE	Max	Min	Mean ± SE	Max	Min
NOF	8.72 ± 1.48	36	1	7.77 ± 1.88	30	1	9.94 ± 2.41	36	2
TDF (m)	1126.81 ± 353.50	9468	8.25	1502.01 ± 582.75	9468	9.75	641.25 ± 279.15	4144.50	8.25
LSF (m)	454.75 ± 124.10	2841	3.75	586.19 ± 200.06	2841	3.75	284.65 ± 112.81	1543.50	4.50
FD (min)	33.46 ± 9.91	255.29	0.24	42.46 ± 16.03	255.29	0.24	21.82 ± 9.15	142.42	0.29
AS (km/h)	1.77 ± 0.06	2.77	1.10	1.88 ± 0.09	2.77	1.30	1.63 ± 0.08	2.26	1.10
MAXS (km/h)	2.87 ± 0.10	5.40	1.90	3.05 ± 0.17	5.40	2.06	2.64 ± 0.11	3.60	1.10

**Table 7:** Summary of flight parameters for *M. galloprovincialis* adults.

Parameters	Total (*n* = 29)			Males (*n* = 15)			Females (*n* = 14)		
	Mean ± SE	Max	Min	Mean ± SE	Max	Min	Mean ± SE	Max	Min
NOF	3.72 ± 0.75	19	1	2.95 ± 1.04	19	1	4.71 ± 1.10	15	1
TDF (m)	538.43 ± 114.40	2774.81	4.02	473.54 ± 170.63	2774.81	10.05	622.39 ± 154.82	1983.87	4.02
LSF (m)	316.04 ± 61.04	1629.11	4.02	269.93 ± 74.61	1112.54	6.03	375.69 ± 102.64	1629.11	4.02
FD (min)	9.46 ± 9.91	143.97	0.09	5.45 ± 1.91	32.22	0.23	14.66 ± 0.26	143.97	2.47
AS (km/h)	2.93 ± 0.16	5.91	2.33	2.71 ± 0.21	5.91	2.33	3.21 ± 0.43	5.91	3.55
MAXS (km/h)	4.82 ± 0.27	8.87	3.55	4.38 ± 0.36	8.87	3.95	5.39 ± 8.94	8.87	0.09
